# Time Interval from Symptom Onset to Hospital Care in Patients with Acute Heart Failure: A Report from the Tokyo Cardiac Care Unit Network Emergency Medical Service Database

**DOI:** 10.1371/journal.pone.0142017

**Published:** 2015-11-12

**Authors:** Yasuyuki Shiraishi, Shun Kohsaka, Kazumasa Harada, Tetsuro Sakai, Atsutoshi Takagi, Takamichi Miyamoto, Kiyoshi Iida, Shuzou Tanimoto, Keiichi Fukuda, Ken Nagao, Naoki Sato, Morimasa Takayama, Scientific Committee of Tokyo CCU Network

**Affiliations:** 1 Tokyo CCU Network Scientific Committee, Tokyo, Japan; 2 Department of Cardiology, Keio University School of Medicine, Tokyo, Japan; Osaka University Graduate School of Medicine, JAPAN

## Abstract

**Aims:**

There seems to be two distinct patterns in the presentation of acute heart failure (AHF) patients; early- vs. gradual-onset. However, whether time-dependent relationship exists in outcomes of patients with AHF remains unclear.

**Methods:**

The Tokyo Cardiac Care Unit Network Database prospectively collects information of emergency admissions via EMS service to acute cardiac care facilities from 67 participating hospitals in the Tokyo metropolitan area. Between 2009 and 2011, a total of 3811 AHF patients were registered. The documentation of symptom onset time was mandated by the on-site ambulance team. We divided the patients into two groups according to the median onset-to-hospitalization (OH) time for those patients (2h); early- (presenting ≤2h after symptom onset) vs. gradual-onset (late) group (>2h). The primary outcome was in-hospital mortality.

**Results:**

The early OH group had more urgent presentation, as demonstrated by a higher systolic blood pressure (SBP), respiratory rate, and higher incidence of pulmonary congestion (48.6% vs. 41.6%; *P*<0.001); whereas medical comorbidities such as stroke (10.8% vs. 7.9%; *P*<0.001) and atrial fibrillation (30.0% vs. 26.0%; *P*<0.001) were more frequently seen in the late OH group. Overall, 242 (6.5%) patients died during hospitalization. Notably, a shorter OH time was associated with a better in-hospital mortality rate (odds ratio, 0.71; 95% confidence interval, 0.51−0.99; *P* = 0.043).

**Conclusions:**

Early-onset patients had rather typical AHF presentations (e.g., higher SBP or pulmonary congestion) but had a better in-hospital outcome compared to gradual-onset patients.

## Introduction

Heart failure (HF) is one of the most frequently encountered cardiovascular conditions [1–3]. Patients with acute heart failure (AHF) present differently; some with abrupt onset of symptoms and others with symptoms that began days to weeks before seeking medical care. Depending on their clinical presentation, there may be a distinct difference in the pathophysiological process [4–7]. However, there is currently little guidance on the manner in which the time interval between AHF symptom onset and hospital admission should be considered by the treating physicians, and its prognostic implications remain unknown.

In addition, the time interval between symptom onset and initial medical care is known to be an important factor for the improvement of the patients’ prognoses in acute coronary syndrome, typically in ST-elevation myocardial infarction; both symptom-to-reperfusion and door-to-balloon times have been demonstrated to be important predictors of ST-segment elevation myocardial infarction outcomes [8,9].

Therefore, in the present study, we aimed to evaluate whether the time interval of symptom-onset to hospital care would affect in-hospital mortality in AHF patients. The dataset of AHF patients admitted to hospitals belonging to the Tokyo Cardiac Care Unit Network (TCN; with 67 institutions) were analyzed. We hypothesized that identification of a clinical cutoff of the time interval between symptom onset and hospital care may aid in improving risk stratification of these patients [10–13].

## Methods

### Study design

The TCN database is an ongoing multicenter registry that prospectively collects information on emergency admissions to acute cardiac facilities via emergency medical service (EMS), and aims to describe the demographic and clinical characteristics of patients hospitalized with acute cardiovascular diseases [14]. Each TCN hospital is accredited by the Metropolitan Tokyo Government and participates in the Tokyo citywide system of acute cardiac care (acute myocardial infarction, unstable angina, arrhythmia, acute heart failure, aortic dissection, and pulmonary embolism). The paramedics, similarly to the Western system, provide medical care at an advanced life support level though they have some limitations (e.g., unable to use non-invasive positive airway pressure ventilation [NIPPV] and administer drugs to patients except for adrenalin in case of cardiopulmonary arrest).

The patient data for TCN is prospectively collected. The EMS members are also mandated to document a series of EMS times of the symptom onset, call receipt, vehicle arrival at the scene, and contact with patients and hospital arrival based on the clock used by each EMS system. These data are integrated into the CCU network registry system sponsored by the Tokyo Metropolitan Government. All data were logically checked by the data manager and were confirmed by the scientific committee.

All patients admitted to cardiac care facilities whose information had been catalogued by the TCN were eligible for participation in this study. By June 2015, 72 hospitals, serving a population of 1.3 million individuals in the metropolitan Tokyo area, had been included in the TCN registry. Informed consent was not required for participation in this study, as all data were anonymously catalogued. Data collection was performed via individual chart reviews. The study protocol was approved by the Institutional Review Board, Keio University School of Medicine, and conducted in accordance with the Declaration of Helsinki.

### Patient and data collection

**Fig 1:**
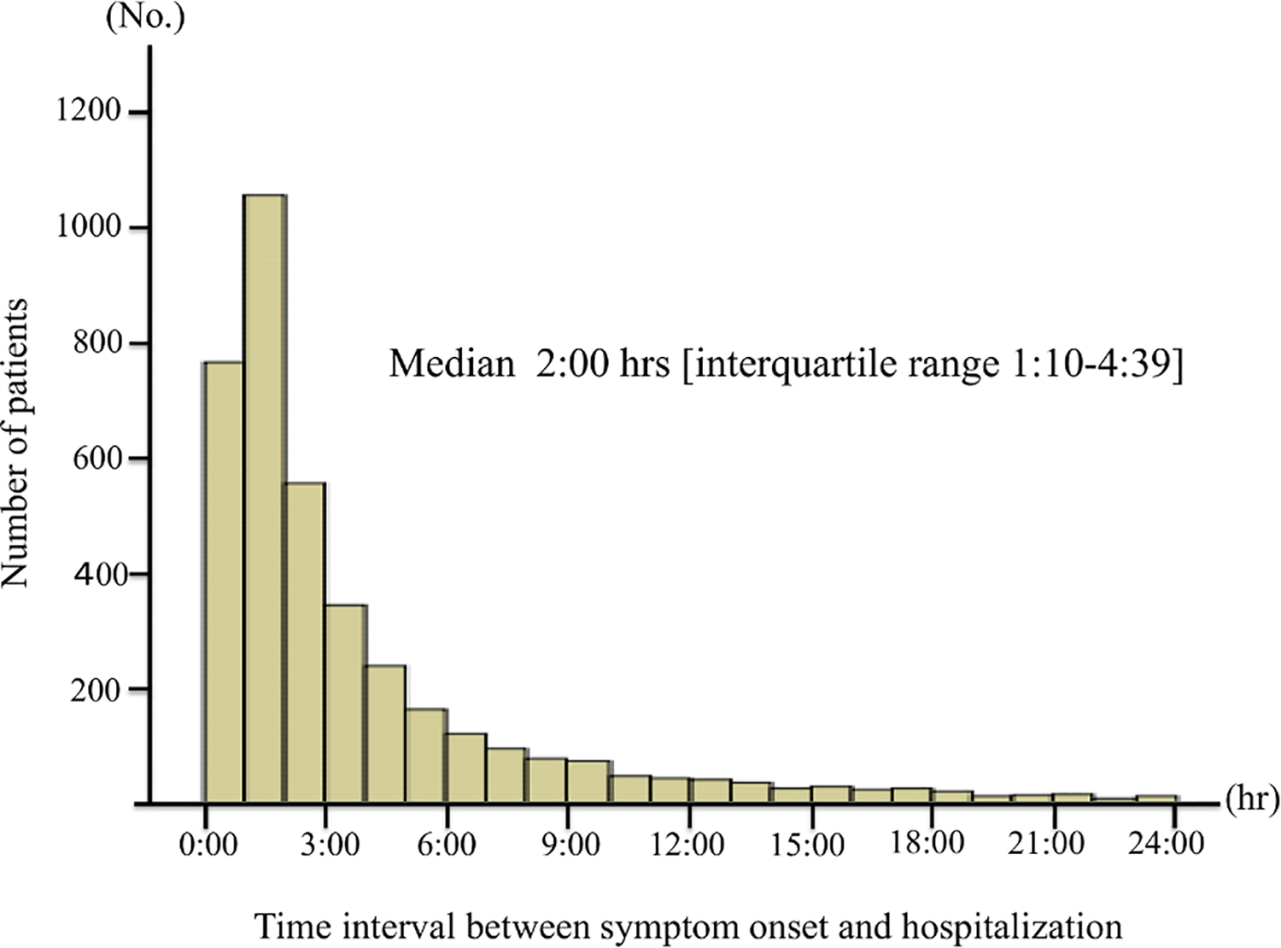
Distribution of onset-to-hospitalization (OH) time. The median of OH time was 2 hours in AHF patients transferred through an ambulance.

**Fig 2:**
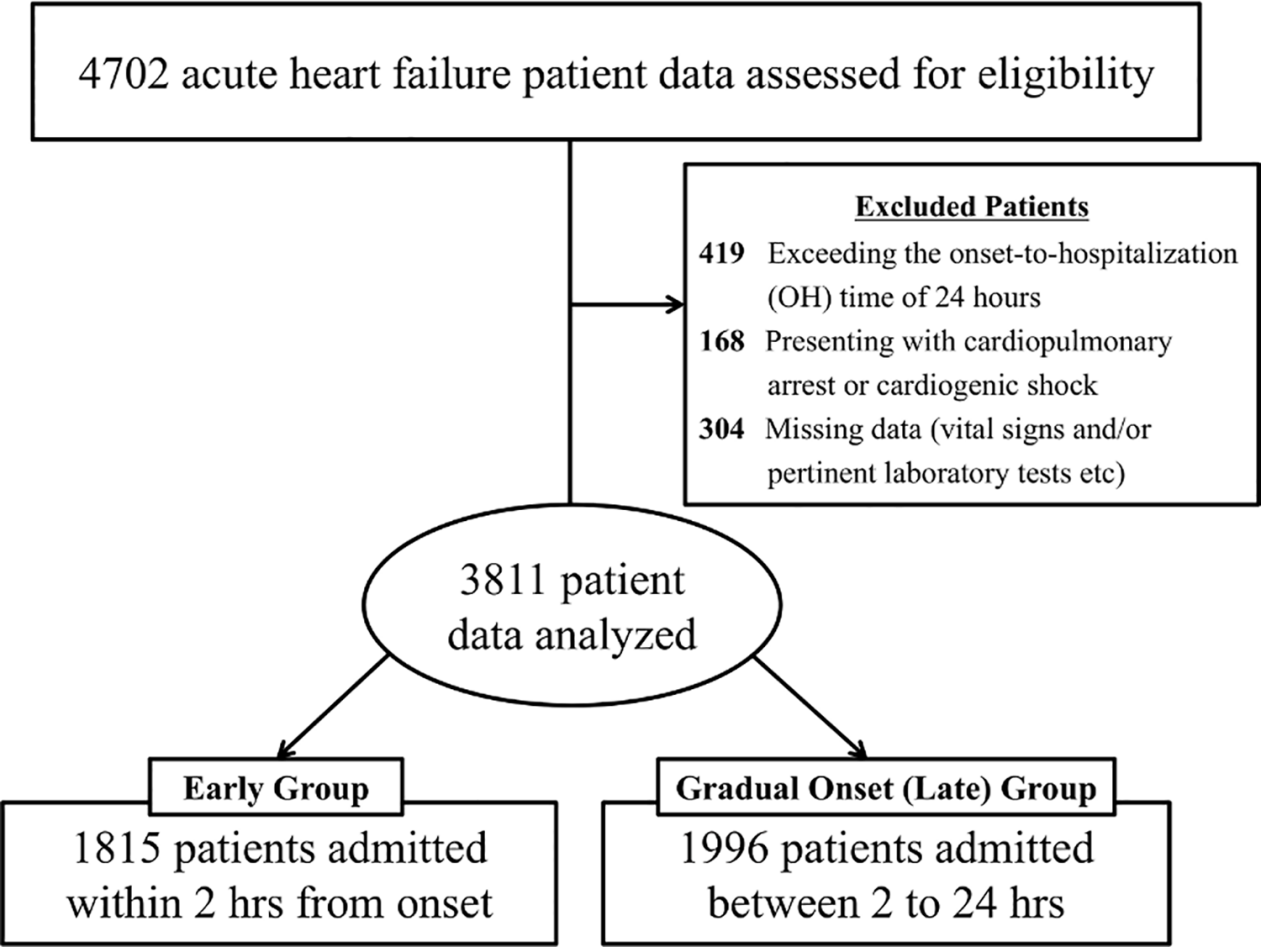
Patient allocation flow chart.

For the present analysis, consecutive patients registered onto AHF dataset was extracted from TCN; the patients that presented with acute coronary syndrome were registered onto separate dataset and not included in the present dataset. From 2009 to 2011, 4702 AHF patients (transferred through EMS) were identified. AHF was defined as rapid-onset HF or a change in the signs and symptoms of HF requiring urgent therapy and hospitalization. Onset-to-hospitalization (OH) time was defined as the time interval between this ‘symptom onset’ and hospitalization. Its distribution is illustrated in Fig 1. Patients with cardiogenic shock, out-of-hospital cardiac arrest (N = 168; 3.9%) or missing vital data (N = 304; 7.0%) were excluded. In addition, the patients transferred from outside of Tokyo Prefecture, and those with an OH time >24 h (N = 419; 8.9%) were also excluded (Fig 2).

The following clinical data on AHF patients were collected: 1) initial vital signs and symptoms in the emergency department (e.g., blood pressure, heart rate, respiratory rate, percutaneous oxygen saturation [SpO_2_], consciousness level, pulmonary congestion according to Killip classification); 2) patient demographics and medical histories; 3) laboratory data; 4) a left ventricular ejection fraction (LVEF); 5) requirements for acute care for heart failure, including assisted ventilation, circulatory support, temporary pacing, and dialysis; and 6) in-hospital mortality rate. We calculated the estimated glomerular filtration rate (eGFR) using the equation defined by the Japan Association of Chronic Kidney Disease Initiative. Anemia was defined as hemoglobin levels <13 g/dl for men and <12 g/dl for women according to the World Health Organization criteria. Consciousness level was assessed using the Japan Coma Scale, which is a simple method for grading neurological disturbance focusing on the patients’ awareness [15,16]. (Table A in S1 File)

### Statistical analysis

The results are expressed as mean ± standard deviation or median with first to third interquartile range (IQR) for continuous variables and as percentages for categorical variables, as appropriate. Statistical comparisons were performed using unpaired *t* tests or the Mann-Whitney test for continuous variables and the Pearson χ^2^ test for categorical variables. Stepwise multiple logistic regression analysis was performed to predict the in-hospital mortality rate. With regard to continuous variables in the multiple logistic regression models, conformity to a linear gradient was graphically assessed. Before multiple logistic regression analyses were performed, multicollinearity was assessed and factors indicating serious multicollinearity were accordingly eliminate from the model. Candidate covariates entered in the model included baseline characteristics with <10% missing values and a univariate association with in-hospital mortality at *P* ≤ 0.10. The stability of the model was assessed every time a variable was eliminated until all statistically non-significant variables (*P* > 0.10) had been eliminated from the model. All probability values were 2-tailed, and *P* values of <0.05 were considered to be statistically significant. All statistical analyses were performed with SPSS version 19.0 (SPSS Japan, Tokyo, Japan).

## Results

### Patient characteristics

Baseline characteristics of the study patients (N = 3,811) are summarized in Table 1. The patients were predominantly male (54.8%), with an average age of 76.3 ± 12.3 years, and an average LVEF of 43% ± 16%. In accordance with prior studies [17], the median OH time (2 h) was used as the cutoff to define early and gradual-onset (late) patients. There were 1815 and 1996 patients in the early (≤2 h; 47.6%) and late (>2 h; 52.4%) groups, respectively.

**Table 1:** Baseline characteristics according to onset-to-hospitalization (OH) time.

	Whole Population	≤ 2 h	> 2 h	*P* value
Variable	(N = 3811)	(N = 1815)	(N = 1996)	
Demographic findings and EMS information				
Age, years	76.3 ± 12.3	76.4 ± 12.0	76.1 ± 12.5	0.472
Male, %	54.8	55.4	54.3	0.498
Body mass index, kg/m^2^	22.3 ± 4.2	22.3 ± 4.3	22.2 ± 4.3	0.401
Scene time, minutes	18 (14−23)	17 (14−21)	19 (15−25)	0.069
Transportation time, minutes	9 (7−13)	9 (6−12)	10 (7−14)	0.178
Clinical findings				
SBP, mmHg	155 (130−181)	160 (135−190)	148 (126−175)	<0.001
SBP< 100 mmHg, %	3.5	2.9	4.0	0.073
SBP 100−140 mmHg, %	33.0	28.0	37.7	<0.001
SBP> 140 mmHg, %	63.5	69.1	58.3	<0.001
DBP, mmHg	87 (70−102)	90 (72−107)	84 (70−100)	<0.001
Pulse rate, beat per minutes	104 ± 27	107 ± 28	101 ± 27	<0.001
Respiratory rate, rate per minutes	26 ± 8	27 ± 8	26 ± 8	<0.001
SpO_2_, %	93 ± 8	92 ± 9	94 ± 7	<0.001
Altered mental status, %	35.3	41.3	30.0	<0.001
Pulmonary congestion, %*	44.8	48.6	41.6	<0.001
Etiology of heart failure				
Ischemic heart disease, %	31.6	33.8	29.5	0.007
Cardiomyopathy, %	13.1	11.7	14.4	0.017
Hypertensive heart disease, %	24.4	25.0	23.8	0.386
Valvular heart disease, %	21.3	21.2	21.5	0.845
Medical history				
Hypertension, %	38.9	39.8	38.1	0.281
Dyslipidemia, %	22.9	21.8	23.8	0.146
Diabetes, %	30.4	30.8	30.0	0.574
Stroke/TIA, %	9.4	7.9	10.8	0.003
Prior admissions of heart failure, %	25.9	25.7	26.2	0.737
Atrial fibrillation/flatter, %	28.1	26.0	30.0	0.009
COPD, %	5.7	5.7	5.7	0.958
Echocardiography				
EF, %	43 ± 16	43 ± 16	43 ± 16	0.771
EF< 40%, %	43.9	44.2	43.5	0.671
Laboratory values				
Hemoglobin, g/dL	11.9 ± 2.5	12.0 ± 2.5	11.8 ± 2.4	0.061
Creatinine, mg/dL	1.1 (0.8−1.7)	1.1 (0.8−1.7)	1.1 (0.8−1.7)	0.385
eGFR, mL/min/1.73m^2^	46.4 ± 26.5	45.8 ± 25.7	46.9 ± 27.3	0.234
Uric acid, mg/dL	6.9 ± 2.3	6.8 ± 2.3	6.9 ± 2.3	0.461
Glucose, mg/dL	188 ± 90	201 ± 94	177 ± 85	<0.001
HbA1c, %	5.9 ± 1.1	5.9 ± 1.1	5.9 ± 1.1	0.432
Total cholesterol, mg/dL	173 ± 46	176 ± 47	171 ± 45	0.017
BNP, pg/mL	781 (400−1411)	782 (400−1382)	781 (399−1455)	0.674
Medical therapy				
Circulatory device, %	2.6	2.6	2.6	0.997
Assisted ventilation, %†	32.7	36.8	29.1	<0.001
Dialysis, %	5.8	5.7	6.0	0.672
Temporary pacing, %	1.5	1.4	1.5	0.801
Duration of admission				
CCU stay, days,	3 (2−6)	3 (2−5)	3 (2−6)	0.002
Hospital stay, days	15 (9−25)	14 (8−24)	15 (9−25)	0.004

Abbreviations: SBP; systolic blood pressure, DBP; diastolic blood pressure, TIA; transient ischemic attack, COPD; chronic obstructive pulmonary disease, EF; ejection fraction, eGFR; estimated glomerular filtration rate, BNP; B-type natriuretic peptide; Data are presented as mean ± standard deviation, median (interquartile range), or percentage, as appropriate.

* Pulmonary congestion is defined as presenting with rales in >50% of each lung or pulmonary edema on chest X-ray according to Killip classification.

† Assisted ventilation includes both endotracheal and non-invasive mechanical ventilation.

### Comparison between the early and late onset-to-hospitalization groups

No significant differences in the majority of the patient demographics including age and gender were noted between both groups. However, systolic blood pressure (SBP) was higher in the early group compared to that in the late group (160 [135–190] vs. 148 [126–175] mmHg, respectively; P <0.001).

Upon arrival to the emergency department, patients in the early group had more typical AHF clinical profile. They had worse respiratory status, as demonstrated by a higher respiratory rate (27/min vs. 26/min), lower SpO_2_ (92% vs. 94%), and altered mental status (41.3% vs. 30.0%). They also had pulmonary congestion represented by Killip classification II and III (48.6% vs. 41.6% in the late group); subsequently, assisted ventilation was more frequently required in the early group (36.8% vs. 29.1%). With regard to the medical histories, a history of stroke or transient ischemic attack and atrial fibrillation or atrial flutter was more frequently noted in the late group. The proportion of patients who had prior admissions for heart failure was similar between both groups.

### Onset-to-hospitalization time and in-hospital mortality

Overall, 242 (6.5%) patients died during their hospitalization (101 [5.7%] and 141 [7.3%] of patients in the early and late groups, respectively), with a shorter OH time being significantly associated with a favorable in-hospital mortality rate in the univariate logistic regression analysis (odds ratio [OR], 0.77; 95% confidence interval [CI], 0.59–1.00; *P* = 0.049). After adjustment for age, sex, and other significant variables (SBP ≤ 140 mmHg, altered mental status, prior admissions for heart failure, chronic obstructive pulmonary disease, anemia, eGFR, assisted ventilation use), a shorter OH time remained significantly associated with a better in-hospital mortality rate compared to the late OH group (OR, 0.72; 95% CI, 0.52−0.99; *P* = 0.043) (Table 2).

**Table 2:** Predictors of in-hospital mortality.

	Univariate Analysis		Multivariate Analysis	
	OR (95% CI)	*P* Value	OR (95% CI)	*P* Value
**Age (10 year-increments)**	**1.33 (1.18−1.51)**	**< .001**	**1.30 (1.11−1.53)**	**0.001**
**Male**	**0.91 (0.70−1.18)**	**0.47**	**1.06 (0.76−1.48)**	**0.72**
**90< SBP <140mmHg**	**2.67 (2.01−3.55)**	**< .001**	**3.08 (2.24−4.24)**	**< .001**
**Altered mental status**	**1.89 (1.43−2.51)**	**< .001**	**1.93 (1.40−2.66)**	**< .001**
**Prior admissions for heart failure**	**1.49 (1.12−1.96)**	**0.005**		
**COPD**	**1.56 (0.94−2.59)**	**0.084**		
**Anemia**	**2.30 (1.67−3.15)**	**< .001**	**1.55 (1.06−2.29)**	**0.008**
**eGFR (10 mL/min/1.73m** ^**2**^ **decrements)**	**1.18 (1.10−1.25)**	**< .001**	**1.12 (1.05−1.21)**	**0.001**
**Assisted ventilation use**	**2.08 (1.59−2.71)**	**< .001**	**2.13 (1.55−2.95)**	**< .001**
**Onset-to-hospitalization time ≤ 2 h**	**0.77 (0.59−1.00)**	**0.049**	**0.72 (0.52−0.99)**	**0.043**

Abbreviations: OR; odds ratio, CI; confidence interval, SBP; systolic blood pressure, COPD; chronic obstructive pulmonary disease, and eGFR; estimated glomerular filtration rate

## Discussion

By analyzing the EMS database from the metropolitan Tokyo area, our results indicate that patients in the early group, that had OH time less than 2hrs, presented more urgently, represented by higher SBP, respiratory rate, and higher incidence of pulmonary congestion. We also observed that OH time was significantly associated with in-hospital mortality in patients with AHF. The late OH group had more medical comorbidities such as stroke, and were associated with increased risk of in-hospital death.

AHF is known to include two distinctively different clinical entities: 1) acute cardiovascular failure (AVF), defined as an abrupt onset of symptoms for which medical care is sought within minutes to hours; and 2) classic congestive heart failure (CCHF), which is characterized by clinical deterioration over days to weeks [6,7]. The commonly perceived pathophysiological basis of CCHF is fluid accumulation, as seen in patients with AHF. Cotter et al. subsequently proposed the concept of AVF, which is characterized by a transient volume shift from the peripheral veins to the pulmonary circulation with no, or slight, fluid accumulation [6,7]. Our results suggest that recognition of OH time may aid in differentiating these two subtypes of AHF; we speculate that the early OH group included a high number of patients with a clinically consistent characteristics of AVF, such as higher blood pressure and, apparently, pulmonary edema on chest X-ray. Further, the differences in the in-hospital mortality rates between the two groups may be, at least partly, due to continuous fluid accumulation, which is typically seen in patients with CCHF, and would lead to sustained activations of neurohormonal factors that likely impair short-term mortality in the late OH group [18].

Consideration of OH time may also have therapeutic implications. The treatments of AHF differ in each individual case, and the OH time may aid in choosing the most appropriate treatment strategy (i.e., vasodilators vs. diuretics). Although it has been previously reported that the early intervention may be effective to improve clinical outcomes of AHF patients[19–23], there is currently no robust evidence that the use of diuretics and vasodilators in AHF relieves dyspnea and improves other clinical outcomes [24,25]. Identifying patients feasible for vasodilators in various aspects is important to improve AHF patients’ prognoses. In addition, acute airway management is essential to consider the treatment of AHF patients, and they need to be applied relatively early during the course of management. Consideration of OH time may contribute to the decision making about the application of a non-invasive ventilation system.

Importantly, querying patients on the onset of the symptoms is mandatory in history taking of AHF patients, and a fundamental process of patient evaluation; thus it is universally applicable. Further studies are warranted to clarify the role of OH time in these previously proposed risk stratification schemes and to assess its therapeutic implications.

AHF remains a significant problem that requires frequent admissions and high mortality rates, and it is important to discriminate high-risk cases from very low-risk cases in the acute settings. To our knowledge, this is the first study to demonstrate that the time interval between symptom onset and hospital admission for AHF possesses a prognostic significance. Although several effective risk stratification models in the acute setting of heart failure have been developed, OH time, or the time interval between the symptom onset, admission to the emergency department, and initial intervention, has not been considered. For example, Fonarow et al. established an effective risk model added into SBP, blood urea nitrogen, and serum creatinine levels that predicts in-hospital mortality in AHF patients [10]. Although the model enables classification of AHF patients into low-, intermediate-, and high-risk groups, it does not include prehospital information that is substantially important for AHF patients.

Risk models including prehospital information such as OH time may provide additional value in the assessment of the patients conditions and risks, which may be vital for improving patients’ prognosis. In addition, the present study was unable to elucidate the impact of the OH time on post-discharge outcomes. Further research are needed in this area to confirm our hypothesis and evaluate whether early heart failure treatment can be decrease post-discharge mortality and readmission rates in patients with AHF. Mebazaa et al. proposed clinical scenarios according to SBP upon presentation, which was found to be well associated with patient characteristics and prognosis [26]. Further, Collins et al. proposed comprehensively how AHF patients should be treated in the emergency department; after an initial triage with high-risk patients (e.g. tachypnea, hypoxia, altered mental status, hypotension and hypoperfusion), the patients must be appropriately dispositioned [27].

The present study of the TCN database is limited by several factors. First, the data were obtained as the result of an observational study, and unmeasured factors may have influenced the clinical outcomes, such as medication before and during hospitalization. Second, this registry is geographically limited to the metropolitan Tokyo area, where dense networks of hospitals and EMS are available, and may not be applicable to other areas of the world or even other areas in Japan, particularly rural areas. Third, there are concerns about the optimal threshold of the time interval between symptom onset and hospitalization. We divided OH time by 10 and noted a trend toward improved prognosis as a shorter OH time persisted (Figure A in S1 File). Therefore, we adopted the median time as being statistically valid in the present study. Fourth, we excluded the patients for who the OH time exceeded 24 hours. Therefore, the patients in the present analysis represented “true acute” heart failure. Finally, we had no information regarding the management in the emergency department. It is possible that the patients with a late OH time waited a long time for medical care and admission (e.g., the time to diagnosis and stabilization of the patients by the emergency physicians or on-duty physicians, consultation with cardiologists, transfer to an inpatient ward). To minimize these confounders, we analyzed the relationship between symptom onset and EMS call. In the limited data from 2524 of AHF patients in this cohort, the time interval between symptom onset and EMS call did not have a statistical significance with in-hospital mortality rate; but a trend toward increased in-hospital mortality was observed (Figure B in S1 File).

## Conclusion

In our study, OH time had significant short-term prognostic implications in AHF patients; a shorter OH time was independently associated with favorable in-hospital mortality rates. The late OH group, albeit presenting with less urgent symptoms, had worse outcomes. Identification of the symptom onset may aid in the precise triage of patients with AHF, including the identification of the pathophysiological process.

## Supporting Information

S1 File Table A. Japan Coma Scale (JCS). **Figure A.** Relationship with onset-to-hospitalization (OH) time and in-hospital mortality. **Figure B.** In-hospital mortality rate according to onset-to-EMS call between the groups.
(DOCX)
