# Supplementary material for: Time Interval from Symptom Onset to Hospital Care in Patients with Acute Heart Failure: A Report from the Tokyo Cardiac Care Unit Network Emergency Medical Service Database
Source: PLoS One. 2015 Nov 12;10(11):e0142017. doi: 10.1371/journal.pone.0142017 (PMC4643062; doi:10.1371/journal.pone.0142017)
Supplement: S1 File — Figure A. Relationship with onset-to-hospitalization (OH) time and in-hospital mortality. Figure B. In-hospital mortality rate according to onset-to-EMS call between the groups. (DOCX) [file pone.0142017.s001.docx]

**~~Supplemental Material~~ Supporting Information**

**~~S1 Appendix~~ Table A. Japan Coma Scale (JCS)**

**~~S2 Appendix~~ Fig A. Relationship with onset-to-hospitalization (OH) time and in-hospital mortality**

**~~S3 Appendix~~ Fig B. In-hospital mortality rate according to onset-to-EMS call between the groups**

**~~S1 Appendix~~ Table A.**

| **Ⅲ. Cannot be roused using any forceful stimuli (expressed in three-digit numbers)**  **(deep coma, coma, semi-coma)** |
| --- |
| **300. Does not respond at all or with change in respiratory rhythm** |
| **200. Responds with slight movements, including decerebrate or decorticate postures** |
| **100. Responds to avoid stimulus** |
| **Ⅱ. Rousable but reverts to previous state if stimulus stops (expressed in two-digit numbers)**  **(stupor, lethargy, hypersomnia, somnolence, drowsiness)** |
| **30. Only by repeated mechanical stimuli** |
| **20. With loud voice or shaking of shoulders; responds with movements or very simple words** |
| **10. Easily by being spoken to; responds with purposeful movements, phrases and words** |
| **Ⅰ. Awake without any stimuli (expressed in one-digit numbers)** |
| **3. Unable to recall name or date of birth** |
| **2. Unable to recognize time, place and person** |
| **1. Fully or almost fully conscious** |
| **Note R: Restlessness, I: Incontinence, A: Apallic state or Akinetic mutism** |

**~~S2 Appendix~~ Fig A.**

We divided the OH time into 10; there was a trend toward improved prognosis with early OH time persisted. Since the numbers for each of these subgroup is fairly limited, the trend was of borderline statistical significance (P=0.102).

**~~S3 Appendix~~ Fig B.**

We divided the time interval between symptom onset and EMS call (onset-to-EMS call: OEMS) into 3 (first tertile, 0:36; second tertile, 1:54). Via a univariate logistic regression analysis, OEMS did not have a statistical significance with in-hospital mortality rate (the number in each group was fairly small); but a trend toward increased in-hospital mortality was observed (second tertile vs. third tertile: OR, 1.35; 95% CI, 0.88−2.08; P = 0.169)**.**
